# Strain Release of 1‐Azabicyclo[1.1.0]butanes as a Gateway to Highly Functionalized Azetidines: New Strategies and Structural Motifs

**DOI:** 10.1002/chem.71087

**Published:** 2026-05-06

**Authors:** Yuri Gelato, Philipp Natho, Marco Colella, Renzo Luisi

**Affiliations:** ^1^ Department of Pharmacy‐Drug Sciences University of Bari “A. Moro” Bari Italy

**Keywords:** 1‐azabicyclo[1.1.0]butanes, azetidines, bioisosteres, spirocycles, technologies

## Abstract

Over the past decade, the interest of synthetic and medicinal chemists in the azetidine ring has continuously risen, given its impact on (positive) modulation of various physicochemical properties. In fact, the exploration of new azetidine‐based monocyclic, spirocyclic, and bridged scaffolds has led to the discovery of new potential 3D bioisosteres for 2D or nonstrained 3D rings frequently employed in drug discovery programs. A popular and recently revived strategy en *route* to azetidines relies on the use of 1‐azabicyclo[1.1.0]butanes as precursors, leveraging the inherent reactivity of the “spring‐loaded” transannular bond, connecting the bridgehead carbon to the nitrogen. This review provides an overview of synthetic advances since 2021, emphasizing newly accessed structural motifs, patterns in activation tactics, and use of enabling technologies such as photocatalysis and flow chemistry.

## Graphical abstract

Over the past decade, the interest in azetidines has continuously risen, by virtue of their highly appealing pharmaceutical properties. Monocyclic, spirocyclic, and bridged azetidines can be accessed by leveraging 1‐azabicyclo[1.1.0]butanes as precursors. In this review, synthetic advances since 2021 have been discussed, putting emphasis on newly accessed structural motifs, patterns in activation tactics, and use of enabling technologies such as photocatalysis and flow chemistry.

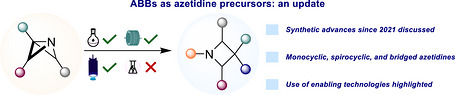

## 1 Introduction

**FIGURE 1:**
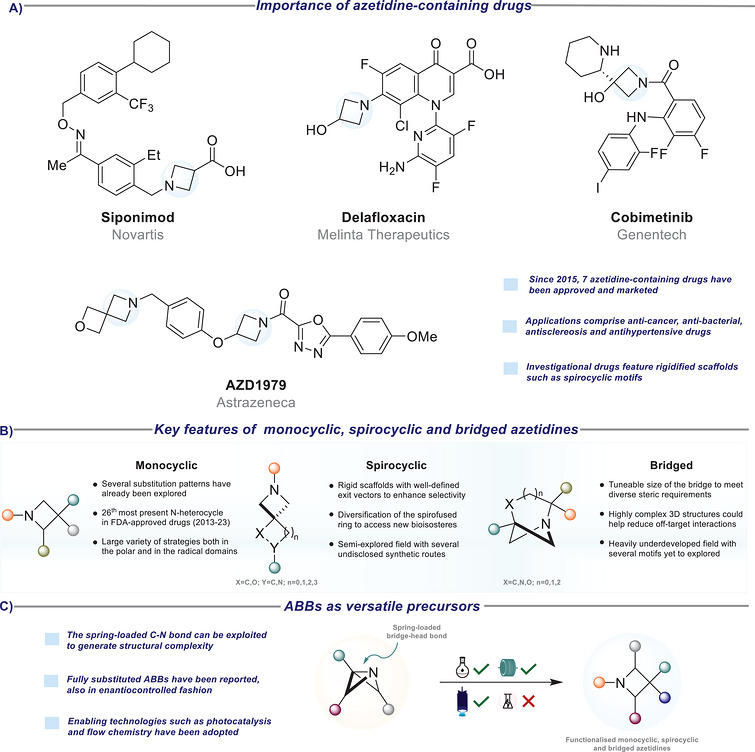
(A) Examples of azetidine‐containing drugs; (B) Key features of monocyclic, spirocyclic, and bridged azetidines; (C) ABBs as versatile precursors.

Since 2015, seven approved small‐molecule drugs incorporate at least one azetidine ring, highlighting an increasing interest around this structural motif. [1] In all cases, 1,3‐substituted azetidines are incorporated, demonstrating the importance of this particular substitution pattern (Figure 1A). The compromise between stability and molecular rigidity makes the (functionalized) azetidine ring a privileged scaffold in drug discovery, allowing the fine‐tuning of pharmacodynamic and pharmacokinetic properties (Figure 1B). Alongside well‐known properties of azetidine‐containing molecules such as improved lipophilicity, solubility, and *in vitro* metabolism, their high F(*sp^3^*) character is receiving growing attention, especially for bioisosteric replacements [2, 3]. Building on the precedence that azetidines can act as bioisosteres of nonstrained *N*‐heterocycles, such as pyrazine, piperidine or pyrrolidine, recent studies have shown that more rigid spirocyclic and bridged motifs, containing the azetidine ring, can be used to this end, too.

Compared to their monocyclic parent compound, azetidine‐bearing spirocycles feature higher structural rigidity and well‐defined exit vectors which allow for the precise orientation of substituents in 3D space, potentially providing enhanced target selectivity and reduced off‐target interactions (Figure 1B) [4]. This is in line with landmark concepts in medicinal chemistry such as *Escape from Flatland*, allowing replacement of planar aromatic rings with their 3D saturated analogues, further pushing interest toward conformational control by consideration of spirocyclic motifs as potential scaffolds for bioisosteric replacement [5, 6]. For example, 2,6‐diazaspiro[3.3]heptane, 2‐oxa‐6‐azaspiro[3.3]heptane, and 1‐oxa‐5‐azaspiro[2.3]hexane have been shown to be bioisosteres of pyrimidine, piperazine, and morpholine, demonstrating the potential of spirocyclic azetidines in medicinal chemistry.

Bridged azetidines, on the other hand, offer unique 3D geometries, allowing for finely‐tuned vectorization of the substituents, as well as functional group shielding, to prevent off‐target interactions [7]. Furthermore, these motifs are characterized by high F(*sp^3^*) character, which is generally associated with lower predicted logP values and, statistically, better chances of clinical success. By varying the bridge size, it is also possible to access scaffolds of several sizes, to meet steric requirements and gain access to new classes of bioisosteres. For example, 1‐azabicyclo[2.1.1]hexanes are proven 3D bioisosteres of pyridines. Despite their appealing properties, the lack of synthetic strategies has heavily limited the adoption of bridged azetidines in investigational drugs. Synthetic advancements toward the accessibility of these motifs are thus imperative.

Although the concept of leveraging 1‐azabicyclo[1.1.0]butanes (ABBs) as synthetic precursors to azetidines is long established, advances in synthetic technology and methodology offer room for synthetic creativity, allowing access to hitherto unexplored interesting chemical space, including a hitherto inaccessible spirocyclic and bridged azetidine‐containing motifs (Figure 1C). In this study, we discuss recent developments in the synthesis of functionalized monocyclic, spirocyclic, and bridged azetidines by exploitation of the high reactivity of the ABB‐bridgehead bond that have been reported since the last exhaustive reviews on this topic [8, 9]. In the timespan between 2021 and 2026, the development of a vast number of protocols has made strain release of ABBs a popular strategy to access azetidines, alongside traditional strategies (*i.e*., ring expansion of aziridines, ring contraction of pyrrolidines, and cyclisation tactics) and recently established ones (*i.e*., aza Paternò−Büchi‐based approaches), as it allows for the concomitant 1,3‐functionalisation of azetidines in one step [10, 11, 12]. We demonstrate that in recent years, new approaches have focused mainly on the introduction of structurally complex substituents to gain access to fully substituted azetidines, also in enantiocontrolled fashion, and to significantly underexplored spirocyclic and bridged azetidines. Furthermore, with a view on driving more sustainable synthetic organic chemistry, enabling technologies such as photocatalysis and flow chemistry were leveraged to grant access to unprecedented substitution patterns.

## 2 Synthesis of Functionalized Monocyclic Azetidines

**SCHEME 1:**
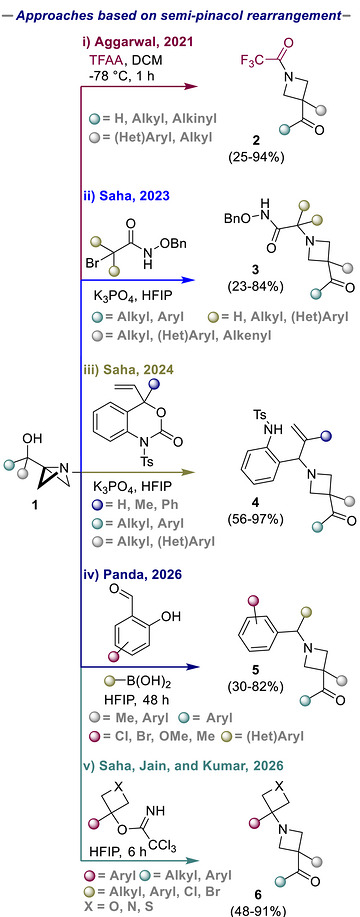
Approaches based on semi‐pinacol rearrangement.

Synthesis of azetidines bearing a quaternary center at C3 is highly pursued, due to the privileged nature of such structural motif in pharmaceutical chemistry, as demonstrated by the range of azetidine‐containing drugs shown in Figure 1A. One of the most popular strategies to access these species exploits semi‐pinacol rearrangement of 1‐azabicyclo[1.1.0]butanes bearing a secondary or tertiary alcohol at C3 (**1**), accessible by nucleophilic addition of (1‐azabicyclo[1.1.0]butan‐3‐yl)lithium to aldehydes or ketones (Scheme 1) [13]. For example, Aggarwal and co‐workers reported that the desired semi‐pinacol rearrangement can be promoted by activation of 1‐azabicyclo[1.1.0]butyl carbinols **1** with trifluoroacetic anhydride affording *N*‐protected C3‐bearing ketones **2** in generally good yields (25%–94%) (Scheme 1i). Both aryl and alkyl migrating groups are tolerated, and expectedly electron‐rich migrating groups lead to higher yields, compared to the electron‐deficient analogues. Notably, when trifluoromethanesulfonic anhydride is used as the activator instead, even deactivated substrates undergo the desired transformation. In fact, highly electron‐deficient 2‐chloropyridine substituent proved to be an inefficient migrating group when trifluoroacetic anhydride was used, but afforded a modest 23% yield of the desired product with trifluoromethanesulfonic anhydride. In a similar vein, Saha and coworkers described the use of aza‐oxyallyl cations as alternative electrophilic activators to promote the desired semi‐pinacol rearrangement of carbinols **1** to ketones **3** (Scheme 1ii)[14]. Such aza‐oxyallyl cations can be generated *in situ* from brominated or chlorinated precursors under basic conditions, and engaged in the activation of ABBs to promote a semi‐pinacol rearrangement, affording densely functionalized products. Interestingly, in addition to variation of the carbinol substituents, structural modification can also be achieved on the aza‐oxyallyl fragment with various aryl and heteroaryl moieties being tolerated. Notably, steric and electronic effects appear to only marginally affect the yields. Building on this work, the same authors also extended the methodology to aza‐ortho‐quinone methides as a new class of electrophilic partners (Scheme 1iii) [15]. Such reactive species can be generated *in situ* from vinyl benzoxazinanone precursors, and used to electrophilically activate ABBs at the nitrogen, to promote strain release *via* semi‐pinacol rearrangement affording *N*‐allylated products **4**. Electron‐rich aryl and heteroaryl residues such as 4‐methoxyphenyl, indolyl, thiophenyl, and 4‐methoxystyryl were competent migrating groups, affording products in generally good yields (56%–97%). Also an enantioselective version of this transformation has been reported [16]. Most recently, a multi‐component Petasis borono‐Mannich reaction has been developed to provide access to highly substituted azetidines **5** (Scheme 1iv) [17]. Specifically, HFIP‐promoted semi‐pinacol rearrangement of ABB **1** affords incipient azetidines which can engage in a Petasis borono‐Mannich reaction with a diverse range of aromatic aldehydes and boronic acids, affording the desired products in 30–75% yield. Notably, an asymmetric version was developed affording the products in >95:5 ee in the presence of chiral (*R*)‐(+)‐3,3′‐dibromo‐BINOL ligand. Leveraging the HFIP‐activation pathway further, Saha, Jain, and Kumar reported a strategy to access a library of azetidines **6** bearing oxetanyl, azetidinyl, or thietanyl substituents at the nitrogen (Scheme 1v) [18]. Furthermore, a catalytic asymmetric strategy to access allylated azetidines was recently reported [19].

**SCHEME 2:**
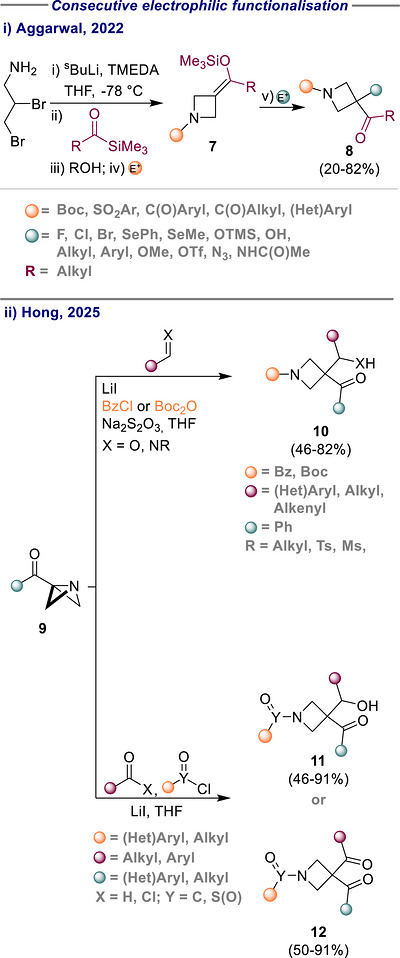
Consecutive electrophilic functionalization.

Overcoming the inherent limitation of semi‐pinacol rearrangement‐based protocols requiring significantly different electronic properties to ensure selectivity, Aggarwal and co‐workers reported a modular approach for the synthesis of structurally diverse azetidines, by means of the consecutive installation of three electrophilic reaction partners (Scheme 2i) [20]. Addition of (1‐azabicyclo[1.1.0]butan‐3‐yl)lithium to acyltrimethylsilane affords an alkoxide which is set up to undergo Brook rearrangement, affording *N*‐protected azetidines bearing a silyl enol ether **7**. Subsequently, exploiting the nucleophilic character of the resulting silyl enol ether species **7**, it is possible to trap a range of electrophiles to access products **8** bearing halides, sulfides, selenides, alcohols, azides, amides, and tertiary amines at C3, in generally good yields.

A related approach was reported by Hong and co‐workers (Scheme 2ii) [21]. It was shown that carbonyl‐substituted 1‐azabicyclo[1.1.0]butanes **9** can be activated by electrophiles such as di‐*tert*‐butyl dicarbonate and acyl chlorides to undergo strain release when treated with lithium iodide, affording 3‐iodo azetidines. Treatment with another equivalent of lithium iodide promotes a redox process which generates a lithium enolate at C3, which can be quenched with electrophiles such as aldehydes, imines and acyl chlorides, affording products **10**–**12** bearing a quaternary carbon. The protocol also lent itself to scale up and late‐stage applications. Whereas a range of electrophiles were shown to effectively promote strain‐release functionalization, alkyl or aryl halides could not be engaged, precluding *N*‐aryl or *N*‐alkyl azetidine synthesis through this protocol.

**SCHEME 3:**
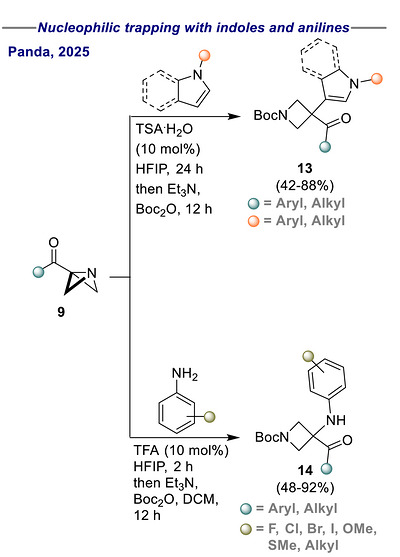
Nucleophilic trapping with heteroaromatics and anilines.

Carbonyl‐substituted ABBs **9** are versatile substrates also for other transformations allowing for diverse substitution pattern on the resulting C3‐quaternary carbon. For example, Brønsted acid catalysis promotes strain release, affording an incipient carbocation on C3, which can be trapped with indole and related heterocycles, including pyrroles, imidazoles, triazoles, and benzotriazoles affording azetidine **13** (Scheme 3) [22]. It is worth noting that anilines are also suitable nucleophiles, in contrast reacting at the nitrogen, thus affording interesting unnatural azetidine‐containing α‐amino ketones **14** in up to 92% yield.

**SCHEME 4:**
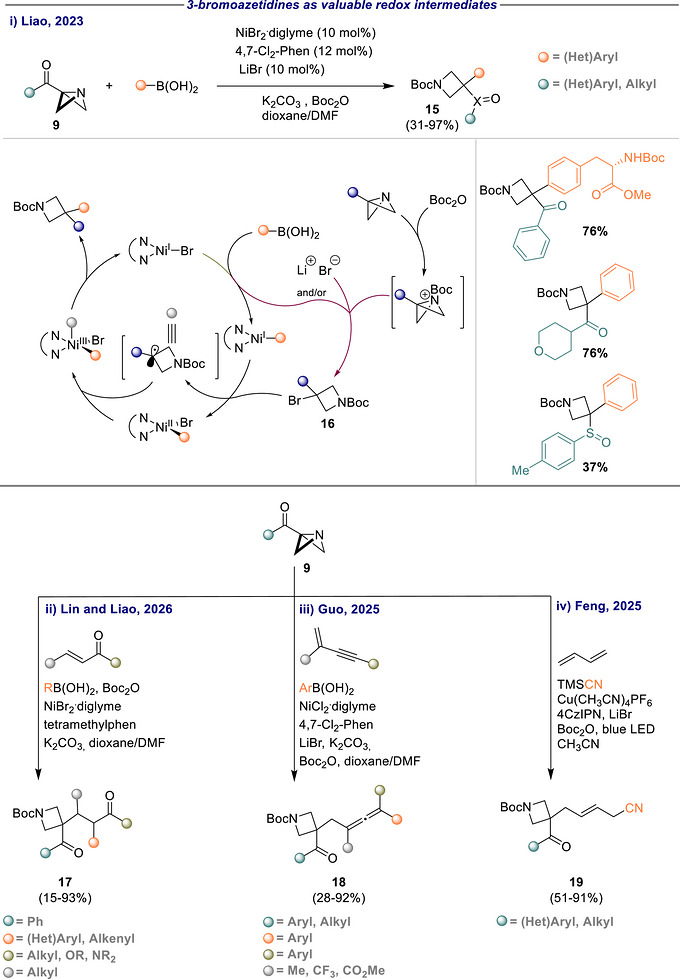
3‐bromoazetidines as valuable redox intermediates.

In addition, also aryl‐ and alkyl‐bearing C3‐quaternary carbons are accessible from the carbonyl‐bearing ABBs **9**, and several transition metal‐catalyzed protocols have been developed. Specifically, Liao and co‐workers reported a nickel‐catalyzed strategy to access C3 arylated or alkenylated products **15**, *via* a Suzuki‐type coupling (Scheme 4i) [23]. Azabicyclo[1.1.0]butanes bearing ketones **9** can be electrophilically activated with di‐*tert*‐butyl dicarbonate, to undergo strain release when treated with lithium bromide. Mechanistically, the resulting 3‐bromoazetidines **16** can then be engaged in Ni‐catalyzed Suzuki‐type couplings. A broad variety of aryl boronic esters proved to be competent reaction partners, allowing for the installation of both electron‐rich and electron‐poor aromatic rings, affording azetidines **15** in generally good yields. In particular, esters, amides, nitriles, ethers, sulfides, ketones, aldehydes, halides, and tertiary amines were all tolerated as substituents of the newly introduced aromatic ring. Notably, a relevant drop in yield was observed when a furane derivative was tested as well as no reactivity with quinoline and pyridine‐based boronic esters.

Recently, further developments of conversion of the same carbonyl‐substituted ABB **9** into 3‐halogenated intermediates as valuable redox intermediates for metal‐catalyzed couplings have been reported. For example, Lin and Liao developed a four‐component strategy (Scheme 4ii) to access highly substituted 1,5‐dicarbonyl compounds **17** by addition to SOMOphiles such as *α,β*‐unsaturated esters, amides, ketones, 1,3‐enynes, and vinylphosphonate esters [24]. The protocol can be extended to aromatic and heteroaromatic boronic esters bearing both electron‐donating and electron‐withdrawing groups, without substantial variations in yields. Notably, in this case pyridine and quinoline‐based boronic esters were tolerated, though with slightly diminished yields. In a similar fashion, allenyl motifs can be installed affording azetidines **18** when the enone is replaced with enynes. (Scheme 4iii) [25]. Leveraging a similar mechanism, a C3 radical intermediate can be generated and trapped with enyne SOMOphiles. After radical addition and conversion of the incipient propargyl radical to an allenyl radical, Ni‐catalyzed Suzuki‐type coupling allows installation of aryl fragments and grants access to fully substituted allenes. Best yields are obtained when the reaction is performed on azabicyclo[1.1.0]butanes bearing ketones at C3, although phenyl‐substituted ABB can also undergo the desired transformation, albeit in reduced yield (38%).

Last, the 3‐bromoazetidine intermediate **16** can also be trapped with dienes and trimethylsilyl cyanide under photoredox conditions affording γ,δ‐unsaturated ketones **19** (Scheme 4iv) [26]. Upon formation of the key 3‐bromoazetidine intermediate **16**, generation of the corresponding radical is enabled by a SET process involving 4CzIPN in its radical anion state. This C3 radical undergoes addition to 1,3‐butadiene, and the incipient allyl radical enters a copper‐catalyzed cycle promoting installation of a cyano‐substituent as a synthetically useful functional handle.

**SCHEME 5:**
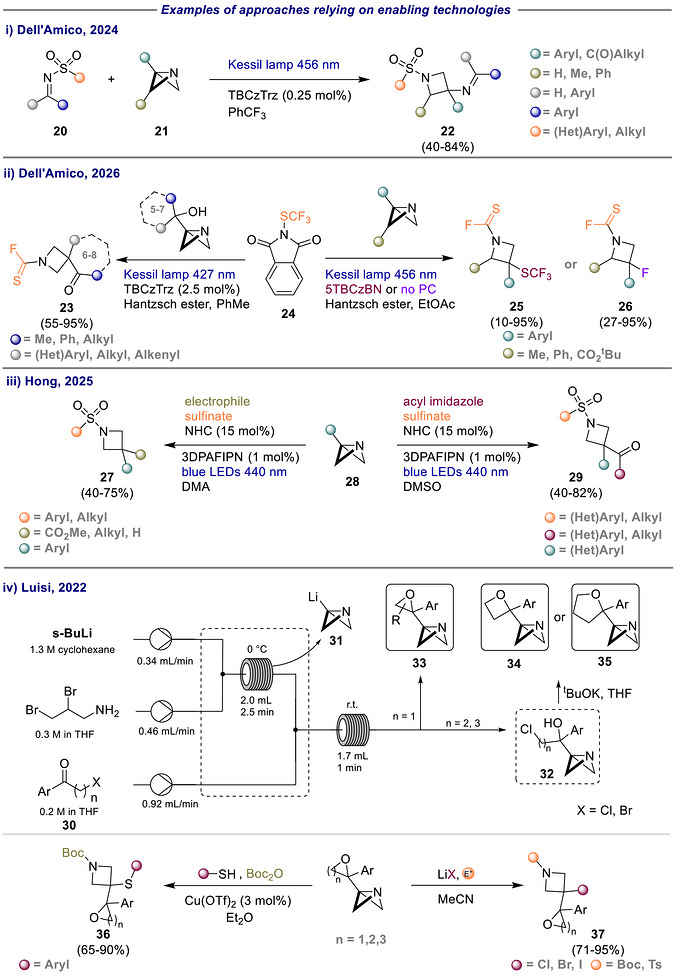
Examples of approaches relying on enabling technologies.

In addition to the latter, several other procedures have been reported in which strain release is achieved through light‐mediated processes. For example, Dell'Amico and coworkers capitalized on energy transfer catalysis to homolytically cleave the labile N‐S bond of sulfonyl imines **20** to generate an electrophilic sulfonyl radical and nucleophilic iminyl radical, which can regioselectively add across the bridgehead bond of ABBs **21**, affording the desired azetidine products **22** (Scheme 5i) [27]. Optimization of the photocatalyst properties proved essential to selectively access the products and prevent undesired iminyl radical dimerization. Furthermore, it was shown that in the presence of an excess of SOMOphiles such as tris(trimethylsilyl)silane (supersilane) and phthalimide‐SCF_3_ **24** it is possible to alternatively install a hydrogen atom or a trifluoromethyl group at C3, instead of the iminyl moiety. Interestingly, these three‐component experiments also led to the formation of thiocarbamoyl fluoride subproducts **23**, **25** and **26**. Careful optimization allowed their selective access by strain release *via* semi‐pinacol rearrangement under photocatalytic conditions (Scheme 5ii) [28]. Several aromatic and heteroaromatic residues proved to be efficient migrating groups for this transformation. Furthermore, replacement of carbinol‐substituted ABBs with aryl‐substituted ABBs allows incorporation of a single fluorine atom **26** or a SCF_3_ group **25** at C3.

A different application of photoredox catalysis was showcased by Hong and co‐workers who reported a conceptually novel approach, achieving an Umpolung transformation to convert nucleophilic sulfinates into electrophilic sulfonyl radicals (Scheme 5iii) [29]. Such species are generated through a single electron transfer process, enabled by 3DPAFIPN in its excited state, and are capable of electrophilically activating aryl‐substituted ABBs **28** at the nitrogen, promoting strain release to generate a C3‐centered radical. Then, *ipso* acylation *via* NHC (nitrogen heterocyclic carbenes) catalysis affording **29** or quenching with alternative electrophiles affording **27**. Electronically and sterically diverse sulfinates can be engaged in the transformation, bearing residues such as 4‐methoxyphenyl, 4‐trifluoromethylphenyl, 4‐pyridinyl, 2‐naphthyl, and cyclopropyl.

Aside from photocatalysis, other enabling technologies have been exploited to access new structural motifs. For example, Luisi and co‐workers reported the use of microfluidic technologies to access unreported azabicyclo[1.1.0]butanes, bearing 3‐, 4‐ and 5‐membered cyclic ethers (**33**‐**35**), which proved suitable azetidine precursors by chlorination (**37**) or copper‐mediated thiolation (**36**) in the presence of a suitable electrophilic activator (Scheme 5iv) [30]. ABB‐Li **31** can be generated in flow and trapped with α‐, β‐, and γ‐haloalkylketones **30**, to access the desired ABBs, either in situ or through a combined flow‐batch protocol under basic conditions in generally good yields (50%–99%). Structural diversity can be achieved at the *O*‐heterocycle, as a variety of aryl and heteroaryl groups is tolerated. Notably, in an extension of this work, also azabicyclo[1.1.0]butyl‐substituted sulfonimidoyl fluorides are tolerated, bridging late‐stage azetidine functionalization with SuFEx chemistry [31].

**SCHEME 6:**
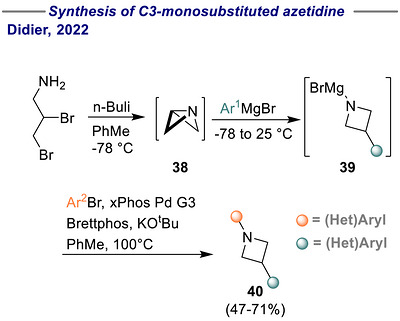
Access to C3‐monosubstituted azetidines.

Whereas all strategies discussed hitherto relied on the synthesis and isolation of C3‐substituted ABBs prior to functionalization, a different strategy was reported by Didier, which relied on the *in situ* synthesis of unsubstituted 1‐azabicyclo[1.1.0]butane **38** (Scheme 6) [32]. One‐pot synthesis of 1‐azabicyclo[1.1.0]butane **38** and treatment with aryl Grignard reagents affords C3‐arylated magnesium amides **39**, which react efficiently with a variety of electrophiles such as *p*‐toluenesulfonyl chloride, di‐*tert*‐butyl dicarbonate, or, substrates suitable for S_N_Ar reactions (e.g., fluorinated pyridines), affording *N*‐ and C3‐bis‐arylated products, in generally good yields (46%–99%). Additionally, to diversify the scope of aryl substituents at the azetidine nitrogen, C3‐arylated NH‐azetidines—arising from Grignard reagents addition at C3 followed by basic hydrolysis—can be engaged in Buchwald‐Hartwig type couplings with electronically diverse aryl bromides bearing substituents such as methoxy‐, trifluoromethyl‐, thioether‐groups, and even formyl‐ and nitro‐groups, as well as heteroarenes such as pyrazines and quinolines, affording *N*‐arylated azetidines **40** in 47–71% yield.

**SCHEME 7:**
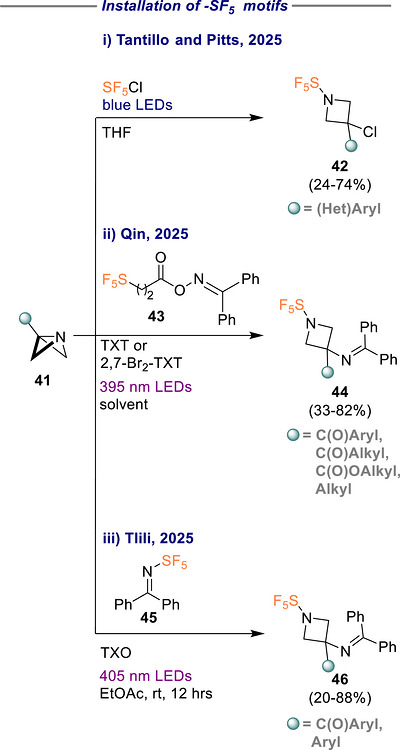
Installation of ‐SF_5_ motifs.

Most recently, azetidines bearing pentafluorosulfanyl groups on the nitrogen have attracted interest of the synthetic chemistry community to gain access to azetidines featuring uncommon substitution patterns. As such, in 2025 several reports elucidated strategies for the synthesis of *N*‐SF_5_ azetidines (Scheme 7) from 3‐substituted ABBs **41** [33, 34, 35]. Tantillo and Pitts reported that, by photoexcitation of SF_5_Cl homolytic cleavage of the sulfur‐chlorine bond affords the corresponding sulfur‐centered radical and a chlorine radical, which can add to ABBs **41** to afford N−SF_5_‐bearing azetidines **42** (Scheme 7i) [33]. Although feasible, the use of sulfur chloride pentafluoride, a highly toxic gas, limits its practical application.

To address this concern, Qin reported a new bench‐stable reagent **43** which, *via* energy transfer catalysis, undergoes controlled collapse to afford a SF_5_ radical and an iminyl radical which regioselectively add across the bridgehead bond (Scheme 7ii) [34]. The protocol can be applied to three classes of ABBs, with slight variations in reaction conditions. In fact, substrates bearing ketones/esters, alkyl‐, or aryl‐substituents at C3 afford products **44** in 33–82% yield. Notably, ketones bearing heterocyclic residues can be employed as well as alkyl chains bearing protected alcohols, with a similar distribution in yield.

The same substitution pattern can be obtained when structurally simpler and easily accessible SF_5_‐bearing imines **45** are leveraged as shelf‐stable SF_5_‐transfer reagents (Scheme 7iii)) [35]. Under energy transfer photocatalysis these undergo homolytic cleavage to transfer both the SF_5_‐group on the nitrogen, as well as an imine‐moiety on the C3 position of azetidine **46**.

**SCHEME 8:**
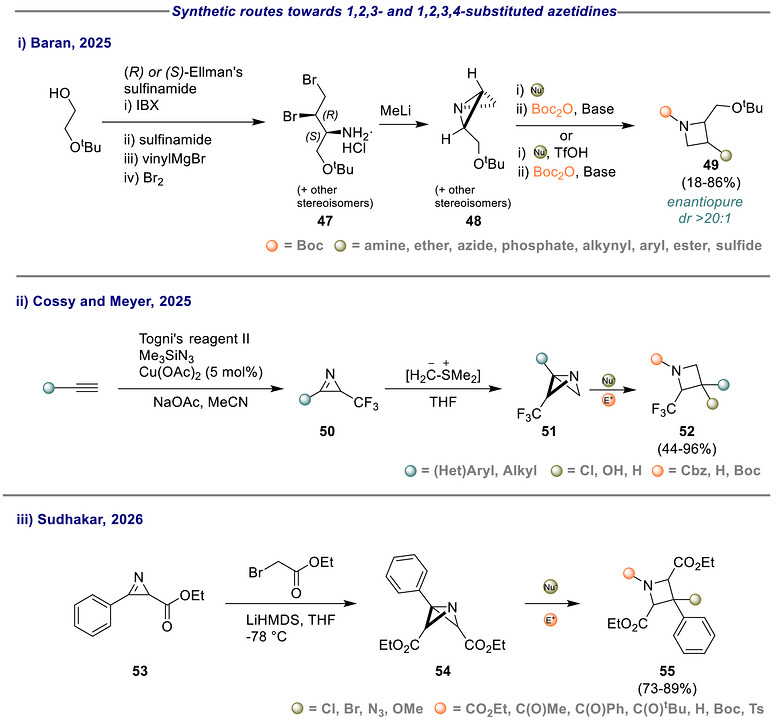
Synthetic routes toward 1,2,3‐ and 1,2,3,4‐substituted azetidines.

Whereas the majority of protocols have focused on the introduction of functionalization across the bridgehead bond, additional C2‐functionalisation remained scarcely explored. In recent years, some reports have begun to pave the way to allow more facile access to functionalized azetidines on multiple sites. For example, Baran reported a strategy to access libraries of 1,2,3‐functionalised enantiopure azetidines, leveraging stereospecific strain release of densely substituted enantiopure ABBs **48**, obtained from twofold intramolecular nucleophilic substitution from substituted amine **47** (Scheme 8i) [36]. A broad variety of nucleophiles comprising alcohols, aromatic heterocycles, azide, phosphates, and thiophenols can be used to promote strain release of 2‐substituted ABBs, through electrophilic activation with di‐*tert*‐butyl dicarbonate toward enantiopure azetidines **49**. The methodology finds application in the synthesis of azetidine stereoprobes which are leveraged in proteomics.

A different study on 1,2,3‐substituted azetidines was devoted to the incorporation of a trifluoromethyl group at C2, considering that the proximity of such group to amines is associated with the modulation of their basicity, and hence the potential reduction of off‐target effects (Scheme 8ii) [37]. In contrast to Baran's approach, the authors relied on the synthesis of 2‐trifluoromethyl‐substituted ABBs **51** through a Johnson‐Corey‐Chaykovsky reaction of azirines **50** bearing the trifluoromethyl moiety. Strain release of the desired ABBs was performed in three different sets of conditions to afford either C3 chlorinated, hydroxylated or hydrogenated products **52**.

Following a similar approach, 1,2,3,4‐substituted azetidines **55** are obtainable through strain release functionalization of 2,3,4‐trisubstituted ABBs **54** (Scheme 8iii) [38]. Such ABBs are accessible through an aza‐Darzens reaction of 2,3‐substituted azirines **53**. The desired 1,3‐functionalisation across the bridgehead bond can be achieved by activation with ethyl chloroformate, acetyl chloride, benzoyl chloride, *tert*‐butyl chloride, or *p*‐toluenesulfonyl chloride. Interestingly, it is possible to access C3‐azido and C3‐methoxy azetidines by treatment of the ABBs **54** with trimethylsilyl azide or methanol, respectively. Notably, in some cases, stereochemical differences in the parent trisubstituted ABB (*i.e*., *syn* vs. *anti*) can lead to drastic variations in yields: specifically, azidation of the anti‐diastereomer afforded the desired product in 81% yield, whereas the same reaction conditions did not afford any product when applied to the syn‐diastereomer.

## 3 Synthesis of Spirocyclic Azetidines

**SCHEME 9:**
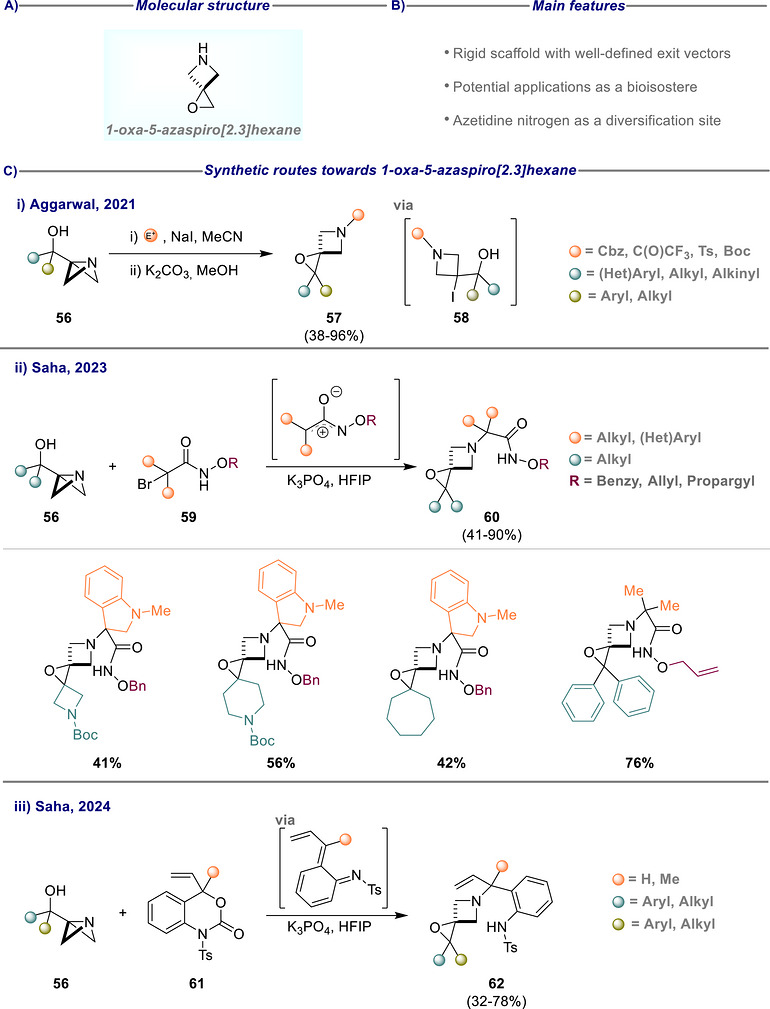
(A) Molecular structure of 1‐oxa‐5‐azaspiro[2.3]hexane; (B) Main features; (C) Synthetic routes.

In recent years, spirocyclic motifs are receiving increasing attention by virtue of their promising pharmaceutical properties. Chemists have valued ABBs as potential precursors and have developed strategies to streamline the synthesis of spirocyclic motifs. To this end, Aggarwal and co‐workers described a strategy to access 1‐oxa‐5‐azaspiro[2.3]hexanes (Scheme 9A)—given their appealing array of properties (Scheme 9B)—through the strain release of azabicyclo[1.1.0]butanes bearing tertiary or secondary alcohols at C3 **56** (Scheme 9C,i). Whereas these substrates efficiently undergo semi‐pinacol rearrangements when promoted by trifluoroacetic or trifluoromethanesulfonic anhydride (*cf*. Scheme 1) [13], replacement of the electrophilic activator with benzyl chloroformate and addition of sodium iodide, diverts reactivity toward the synthesis of 3‐iodoazetidine intermediates **58**, which undergo base‐induced intramolecular cyclisation to spirocycle **57**. It is worth noting that it is possible to synthesize products featuring pyridinyl, indolyl, and propargyl residues as well as dispiro compounds bearing an additional azetidine‐ or cyclohexene‐ring. Other electrophilic activators such as *p*‐toluenesulfonyl chloride or di‐*tert*‐butyl dicarbonate afford products in comparable yields.

Drawing inspiration from this seminal report, Saha and co‐workers describe that also aza‐oxyallyl cations (generated *in situ* from precursor **59**) are suitable electrophilic activators for intramolecular epoxidation—notably, and in contrast to Aggarwal's seminal report, in one step (Scheme 9C,ii) [14]. Whereas semi‐pinacol rearrangement is usually observed under such conditions (*c.f*., Scheme **1**), certain substrates are prone toward the intramolecular epoxidation pathway toward epoxides **60**. This methodology can also be extended to leveraging aza‐*ortho*‐quinones as activators (Scheme 9C,iii) [15]. As such, ABB‐carbinols **56** featuring aliphatic and aromatic substituents can be treated with vinyl benzoxazinanones **61**, in a basic HFIP environment, to afford products **62** in 32–78% yield, although lower efficiency is observed when electron‐withdrawing groups are present on the carbinol.

**SCHEME 10:**
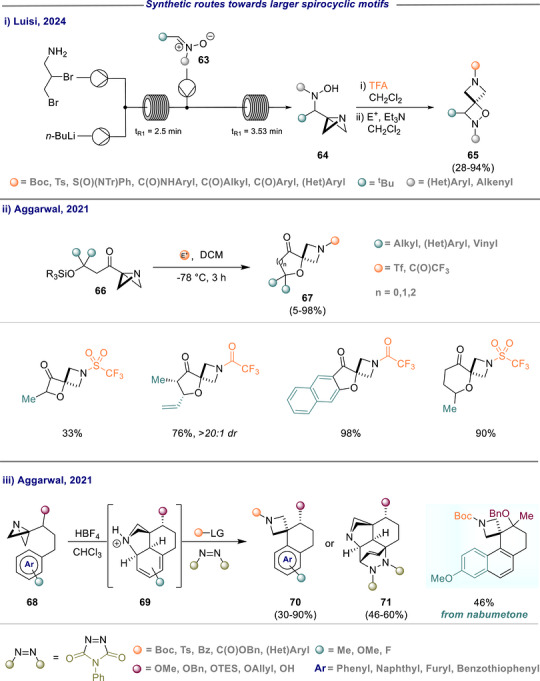
Synthetic routes toward larger spirocyclic motifs.

Intramolecular spirocyclization by strain‐release of the ABB‐moiety is however not limited to the formation of epoxides, but can be extended to larger spirocycles. As such, Luisi and co‐workers reported a combined flow‐batch strategy to access 1‐oxa‐2,6‐diazaspiro[3.3]heptanes **65**, whose potential as a novel piperazine bioisostere was also investigated in silico (Scheme 10i) [39]. With regards to its synthesis, 3‐lithiated ABB is generated under microfluidic conditions and reacted with nitrones **63** to access hydroxylamine‐substituted ABBs **64**. Cyclisation is subsequently promoted by treatment with di‐*tert*‐butyl dicarbonate in the presence of an acid catalyst, affording the desired products **65** in 60–94% yield. In addition to di‐*tert*‐butyl dicarbonate, also trifluoroacetic acid promotes intramolecular cyclisation, allowing further base‐mediated nitrogen functionalization in a subsequent step with sulfonyl chlorides, sulfonimidoyl chlorides, isocyanates, isothiocyanates, and acyl chlorides. The protocol can be extended to acyl chlorides obtained from pharmaceutically relevant structures such as ibuprofen, flurbiprofen, indomethacin, and naproxen. In addition, Buchwald‐Hartwig coupling allows introduction of aryl and heteroaryl motifs comprising pyrazines and indoles, in 28–57% yield.

Beyond this, also strategies to access spirocyclic motifs bearing even larger ring sizes have been explored. Specifically, spirocycles ranging from oxa‐azaspiro[3.3]heptane to oxa‐azaspiro[3.6]decane are accessible leveraging a cyclisation strategy based on the use of ABBs tethered to primary, secondary and tertiary silyl ethers **66** as precursors (Scheme 10ii) [40]. Such species can be prepared by treating ABB‐Li with esters or Weinreb amides. Subsequent treatment with trifluoroacetic or trifluoromethanesulfonic anhydride promotes cyclisation to access spiroazetidines **67** bearing trifluoroacetyl or trifluoromethanesulfonyl groups at the nitrogen. Starting from cyclic silyl ethers, dispirocyclic motifs can be accessed. Although typically yields range from 40–98%, formation of energetically unfavorable oxa‐azaspiro[3.6]decane motif proceeds in a low 5% yield.

Although previously introduced spirocyclisation protocols relied exclusively on the formation of a carbon‐oxygen bond, also carbon‐carbon bond formation has been explored for spirocyclisation. As such, azabicyclo[1.1.0]butane‐tethered (hetero)aryls **68** undergo a tetrafluoroboric acid‐promoted Friedel‐Crafts reaction to afford a congested azabicyclo‐[2.1.1]hexane intermediate **69** stemming from the interaction between the nitrogen of the newly generated azetidine and the electrophilic cationic carbon of the Wheland intermediate (Scheme 10iii) [41]. Upon basic treatment and electrophilic activation with di‐*tert*‐butyl dicarbonate, *p*‐toluenesulfonyl chloride, benzyl chloroformate, benzoyl chloride, or, more interestingly, aromatic halides, the desired aromatic fused azaspiro[3.5]nonane derivatives **70** are obtained. Expectedly, the electronic properties of the aromatic ring are of importance, as electron‐rich arenes such as a 3,5‐dimethoxyphenyl derivative and electron‐poor arenes such as a 3‐fluoro derivative give the corresponding products in markedly different yields (85% vs. 30%, respectively). Additionally, a different reaction pathway is also accessible by trapping the diene portion of the dearomatized intermediates through a Diels‐Alder reactions with 4‐phenyl‐1,2,4‐triazole‐3,5‐dione, affording structurally unique dearomatized scaffolds **71**.

## 4 Synthesis of Bridged Azetidines

**SCHEME 11:**
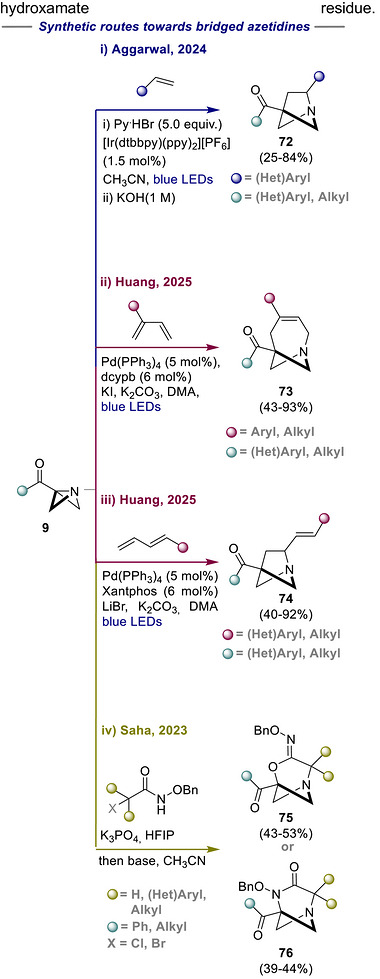
Synthetic routes toward bridged azetidines.

Bridged azetidines are receiving increasing attention because of their complex 3D structures, often associated with improved target selectivity. The Aggarwal group contributed to this field with the first example of formal radical [2σ+2π] cycloaddition featuring ABBs as substrates (Scheme 11i) [42]. The reaction between the bridgehead bond of ABB **9** and an alkene is promoted by pyridine hydrobromide under photochemical conditions using an Iridium‐based photocatalyst. Nonetheless, the transformation relies on an initial polar activation mode, by addition of hydrogen bromide to the N‐C3 bond of the ABB **9**, affording a 3‐bromo azetidine which is then prone to undergo single electron transfer, leading to the formation of a carbon centered radical at C3. Subsequent Giese‐type addition to a styryl partner, oxidation of the incipient benzylic radical to a cation and quenching with the previously liberated bromide, affords benzyl bromide intermediates, which participate in a cyclisation event under basic conditions that leads to the desired products **72**. A range of styryl partners bearing halogens, boronic esters, esters, and amines are tolerated affording the desired products in 25–84% yield.

In a conceptually related approach relying on initial polar halogenation, followed by photochemically initiated formation of a carbon‐centered radical by single electron transfer, also dienes are suitable cycloaddition partners (Scheme 11ii, Scheme 11iii) [43]. Interestingly, the substitution pattern on the diene, as well as small variations in the reaction condition can divert the reactivity into two different outcomes: formal [2+2]‐cycloaddition, affording bridged azetidines **74**, or formal [2+4]‐cycloaddition affording the larger analogues **73**. Specifically, when 1‐aryl dienes are used, a cinnamyl radical is formed, affording 1‐azabicyclo[2.1.1]hexanes **74** through a palladium‐catalyzed cyclisation. In contrast, 2‐aryl dienes undergo a palladium‐catalyzed cyclisation to afford 1‐azabicyclo[4.1.1]octenes **73** via a benzyl‐allyl radical in up to 93% yield. In addition to a wide array of electronically diverse substituted phenyl‐derivatives, notably, also sensitive heterocycles such as thiophene and furane were compatible.

Cyclisation across the bridgehead bond of ABBs is however not limited to alkene partners. Saha demonstrated that also aza‐oxyallyl cations are suitable intermediates in the formation of bridged azetidines (Scheme 11iv) [14]. The authors showed that after the installation of the hydroxamate residue, in situ formed 3‐haloazetidines, are prone to undergo cyclisation, when exposed to basic treatment. Notably, two different classes of products **75** and **76** were observed, based on whether *O*‐cyclisation or *N*‐cyclisation occurred, affording either imidates or amides. Higher selectivity for the *O*‐cyclisation product **75** was usually observed, mainly due to steric effects. Aryl and heteroaryl moieties as well as aliphatic groups are tolerated on the hydroxamate residue.

## 5 Summary and Outlook

Considerable progress has been made in the development of strategies to synthesize functionally complex azetidines from ABBs. A particular focus has been placed on accessing 1,3‐disubstituted azetidines *via* a range of activation modes including semi‐pinacol rearrangement, Brook rearrangement, or transition metal catalysis, allowing access to azetidine substitution patterns which were previously virtually inaccessible. Furthermore, modular approaches have allowed access to 1,2,3‐ and 1,2,3,4‐substituted patterns. Nevertheless, despite the interest and potential of this motif, to date only a limited number of asymmetric approaches exist, and we expect further development in the area in the near future. Within the realm of rigidified scaffolds, significant enhancements were made in the synthesis of pharmaceutically valuable spirocyclic motifs such as 1‐oxa‐5‐azaspiro[2.3]hexane or 1‐oxa‐2,6‐diazaspiro[3.3]heptane demonstrating the growing interest toward such complex 3D structures. Given their potential application as bioisosteres, we expect further progress in the development of such spirocyclic motifs with a view toward more modular substitution patterns, or “angular” azetidines which are currently underexplored. Finally, seminal reports of strain release *en route* to bridged motifs such as 1‐azabicyclo[2.1.1]hexane, 1‐azabicyclo[4.1.1]oct‐3‐ene, and 4‐oxa‐1‐azabicyclo[3.1.1]heptane have shed light on a powerful new approach to the synthesis of this neglected family of azacycles. Last, we have shown the growing adoption of enabling technologies toward strain‐release reactions of ABBs, allowing transformations which are otherwise impossible or challenging under “classical” conditions. Whereas the benefits of photochemistry and continuous flow approaches for strain‐release reactions of ABBs have been demonstrated, the application of other enabling technologies (e.g., electrochemistry or mechanochemistry) is still outstanding and we expect developments in this area in the near future to further expand the accessible chemical space.

## Conflicts of Interest

The authors declare no conflicts of interest.
